# Hierarchical Clustering Using the Arithmetic-Harmonic Cut: Complexity and Experiments

**DOI:** 10.1371/journal.pone.0014067

**Published:** 2010-12-02

**Authors:** Romeo Rizzi, Pritha Mahata, Luke Mathieson, Pablo Moscato

**Affiliations:** 1 Dipartimento di Matematica ed Informatica, University of Udine, Udine, Italy; 2 New South Wales Rural Doctors Network, Newcastle, Australia; 3 Centre for Bioinformatics, Biomarker Discovery & Information Based Medicine, The University of Newcastle, Callaghan, Australia; 4 Information Based Medicine Program, Hunter Medical Research Institute, Newcastle, Australia; Dana-Farber Cancer Institute, United States of America

## Abstract

Clustering, particularly hierarchical clustering, is an important method for understanding and analysing data across a wide variety of knowledge domains with notable utility in systems where the data can be classified in an evolutionary context. This paper introduces a new hierarchical clustering problem defined by a novel objective function we call the *arithmetic-harmonic cut*. We show that the problem of finding such a cut is 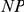-hard and 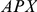-hard but is fixed-parameter tractable, which indicates that although the problem is unlikely to have a polynomial time algorithm (even for approximation), exact parameterized and local search based techniques may produce workable algorithms. To this end, we implement a memetic algorithm for the problem and demonstrate the effectiveness of the arithmetic-harmonic cut on a number of datasets including a cancer type dataset and a corona virus dataset. We show favorable performance compared to currently used hierarchical clustering techniques such as 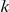-Means, Graclus and Normalized-Cut. The arithmetic-harmonic cut metric overcoming difficulties other hierarchal methods have in representing both intercluster differences and intracluster similarities.

## Introduction

The problem of finding structure in a set of unlabeled data (the so-called *clustering problem*) appears in various domains of research including bioinformatics, machine learning, image processing and video processing. In the area of bioinformatics, clustering has become increasingly important, as finding genetic subtypes of heterogeneous diseases like breast cancer, ovarian cancer and multiple sclerosis, may be made easier by using suitable clustering methods. This work aims to facilitate this line of research by finding good clusterings of various datasets with known partitions.

The importance of the clustering problem in various areas has given rise to several greedy algorithms such as 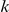-Means, optimization-based methods such as Normalized-Cut and neural-net based methods amongst others.

In this work, we will use a top-down approach for hierarchical clustering, recursively dividing the elements in the data. In each division step, often a graph partitioning technique is used (a similar approach is used for Normalized-Cut [1]). However, many graph (bi)partitioning problems can be formulated as 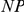-hard optimization problems, for which there are no polynomial-time algorithms to find the optimal solution unless 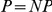. This is an indication of the difficulty of the clustering problem and the focus of research since the work of Wertheimer [2]. In this work, we propose a new objective function for graph bipartitioning. The motivation for finding a new objective function for graph bipartitioning is that the known bipartitioning methods produce incorrect results for some datasets. For example, two formulations for clustering by graph partitioning are Max-Cut [3] and Normalized-Cut [1]. Max-Cut is already known to provide incorrect results for some datasets [1]. We show that Normalized-Cut also produces some incorrect results for some of the datasets examined.

To achieve a better clustering than agglomerative hierarchical clustering and existing graph partitioning formulations, our proposed objective function seeks to minimize the intra-cluster distances and at the same time it seeks to maximize the inter-cluster distances. Our objective function performs well in clustering diverse types of datasets.

More precisely, we pose the hierarchical clustering problem as a finite number of instances of a graph partitioning problem, called Arithmetic-Harmonic Cut (AH-Cut). In the AH-Cut problem, we start with a distance matrix for a set of objects and compute a weighted graph in which vertices represent objects and edges are weighted by the distance between the corresponding vertices. Our objective function tries to obtain a partition where the weight of the partition is directly proportional to the sum of the weights on the edges between the two partite sets and the sum of the reciprocals of the weights on the edges inside the partite sets. When considered as an optimisation problem, the goal is to maximise the weight of the partition. The recursive application of AH-Cut can then be used to generate a tree-based classification of the data.

As noted many graph bipartioniting problems are 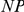-hard at least, so a theoretical examination of any proposed clustering problem is necessary to determine whether it constitutes a practical approach to clustering. We give such a classification of AH-Cut and show that although it is 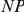-hard and hard to approximate, it is *fixed-parameter tractable*, and therefore still a practical method for clustering.

### Related Objective Functions for Hierarchical Clustering

#### The 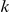-Means Algorithm

The 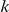-Means algorithm is one of a group of algorithms called *partitioning methods*; Given 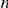 objects in a 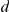-dimensional metric space, we wish to find a partition of the objects into 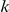 groups, or clusters, such that the objects in a cluster are more similar to each other than to objects in different clusters. The value of 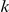 may or may not be specified and a clustering criterion, typically the squared-error criterion, must be adopted. The 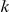-Means algorithm initializes 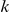 clusters by arbitrarily selecting one object to represent each cluster. Each of the remaining objects are assigned to a cluster and the clustering criterion is used to calculate the cluster mean. These means are used as the new cluster points and each object is reassigned to the cluster that it is most similar to. This continues until there is no longer a change when the clusters are recalculated. However, it is well-known that depending on the initial centres of the clusters, clustering results can change significantly. We use Gene Cluster 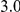 [4] for comparing our method with 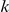-Means.

#### Max Cut, Ratio Cut and Average Cut

Graph bipartitioning algorithms are also used for clustering [5]. Given a graph 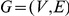 and perhaps a weighting function 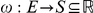, a graph bipartitioning problem asks for a partition 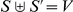 such that some function on the (weights of the) edges between 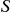 and 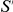 satisfies the given bound, or in the case of an optimisation problem, is optimised. One of the most common formulations is essentially an 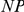-hard combinatorial problem, called Weighted Max-Cut, which is a simple weighted extension of the Max-Cut problem. If we denote the edges between 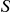 and 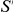 as 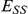 then the function 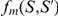 to be optimised in the case of Weighted Max-Cut is:
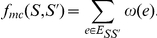
Although good algorithms exist for Weighted Max-Cut, Shi and Malik [1] and Wu and Leahy [5] show that (Weighted) Max-Cut 's objective function favours cutting small sets of isolated nodes in the graph. Furthermore, during bipartitioning, sometimes it may also cut small groups and put two parts of the same small group into different partite sets.

Ratio-Cut uses the objective function:
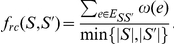
In this case 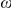 is taken as a similarity metric. Ratio-Cut (and its 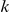-way extension) has also been employed for image segmentation [6] and circuit partitioning for hierarchical VLSI design [7].

Average-Cut employs the following objective funtion:
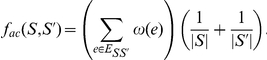
If 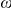 is a similarity metric, the the problem becomes a minimisation problem, 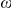 expresses distance the goal is maximisation. It turns out that even using the average cut, one cannot simultaneously minimise the inter-cluster similarity while maximizing the similarity within the groups.

#### Normalized-Cut

In the context of image segmentation, Shi and Malik [1] introduce Normalized-Cut. They use a similarity metric for 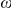, and thus Normalized-Cut is typically expressed as a minimisation problem with the following objective function:
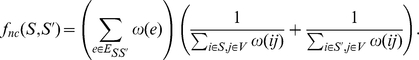



It is well-known that by negating weights the Max-Cut problem is equivalent to the corresponding Min-Cut problem where one is supposed to minimise the sum of the weights (given by some similarity measure) between the two partitions (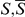) of a set of vertices 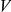 in a graph 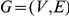. It is straightforward to see that the same argument holds in case of Normalized-Cut as well, which allows the negation of a distance matrix to be used a similarity matrix, facilitating comparisons for datasets for which only distance matrices are available. However, Shi and Malik [1] start with a Euclidian distance matrix 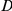 and then compute 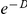 as their similarity matrix. We use both approaches and demonstrate that the performance of this algorithm varies depending on the dataset and the two similarity measures.

Furthermore, Shi an Malik's [1] implementation relaxes the Normalized-Cut problem into a generalised eigen-value problem by allowing the vertices 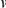 to take real-values, instead of taking values from just the set 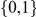 where 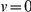 denotes that 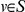 and 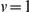 denotes that 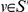. Then, for bipartitioning, the second smallest eigenvector of the generalized eigen system is the real-valued solution to Normalized-Cut. Finally, they search for the splitting point as follows: first choose 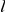 equidistance splitting points, compute Normalized-Cut's objective value for each of these splits, then choose the one for which Normalized-Cut's objective value is the smallest. In fact, the implementation also allows 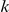-way Normalized-Cut, Yu and Shi [8] examine this further. It considers the first 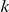 eigen vectors and yields 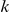 partite sets from a discretisation step following it.

Notice that Max-Cut and Ratio-Cut do not cluster by intra-cluster similarity and this results in a poor clustering results for image segmentation in comparison to Normalized-Cut [1]. Therefore, among these three algorithms, we consider only Normalized-Cut for comparison with our algorithm.

#### Graclus

Graclus [9] implements a multilevel algorithm that directly optimizes various weighted graph clustering objectives, such as the popular ratio cut, normalized cut, etc. This algorithm for multilevel clustering consists of three steps: (a) iteratively merging nodes of the graph (using various criteria of merging) and creating supergraphs with fewer nodes; (b) applying some base-level clustering on the resulting supergraph; and (c) restoring the clusters of original graph iteratively from the clustering of the final supergraph. This algorithm does not use eigenvector computation, and is thus notably faster than existing implementations of normalised and ratio-cuts. However, in most of the examples shown in this paper, Shi and Malik's [1] implementation of Normalized-Cut results in a better clustering than Graclus.

### Outline of the Paper

In this paper, after introducing the problem, we first examine the approximability of AH-Cut. In fact, we prove that AH-Cut is 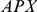-hard (and 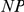-complete) via a reduction from the Max-Cut problem, which is already known to be 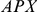-hard [3]. Therefore 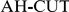 has no polynomial time approximation scheme unless 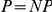. We then demonstrate that AH-Cut is fixed-parameter tractable via a greedy localisation algorithm. Such a complexity analysis provides an indication of what practical methods are suitable for application to the problem. In this case the complexity results indicate that there is unlikely to be a polynomial time algorithm (or even approximation), but that the exponential component of the running time is at worst only a function of a small independent parameter and therefore the problem is likely to still have effective algorithms.

Given the complexity result we use a meta-heuristic approach (namely, a *memetic algorithm*) for AH-Cut (an outline of which was presented previously [10]). We compare the performance of our algorithm on four diverse types of datasets and compare the results with two recent and highly regarded clustering algorithms: Normalized-Cut; and 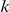-Means. The results indicate that AH-Cut gives a robust and broadly useful hierarchical clustering method.

### Preliminaries

#### Graph Notation and Problem Definition

We consider only simple, undirected graphs, which may or may not be associated with a weight function on the edges. Given a graph 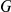 unless otherwise specified we denote the vertex set of 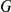 by 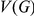 and the edge set of 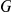 by 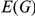. We denote an edge between vertices 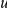 and 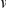 by 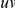.

Given a graph 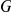 and two vertex sets 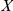 and 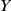 we denote the set of edges with one endpoint in 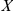 and the other in 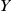 by 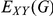. When the graph is clear from context we write 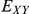.

Arithmetic-Harmonic Cut (AH-Cut)

*Instance:* A graph 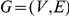, two positive integers 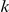 and 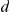 and a weight function 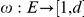.

*Question:* Is there a partition of 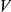 into two sets 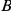 and 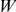 such that
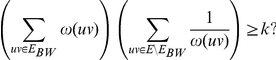

Given a graph 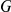 and two disjoint vertex sets 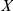 and 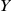, for convenience we denote
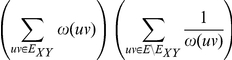
by
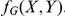
The optimisation verion of AH-Cut is identical except that we maximise the function 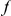.

#### Approximation and Complexity

If a maximisation problem 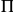 with objective function 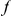 has an polynomial time algorithm which given an instance 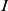 with optimal solution 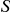 guarantees a solution 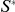 where 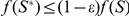 for some 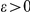 then we say 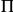 has a *constant factor approximation algorithm*. If there is an algorithm that guarantees such a bound for every 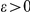, 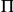 has a *polynomial time approximation scheme* (*ptas*). 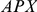 is the class of all problems which have constant factor approximation algorithms. If a problem 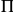 is 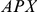-hard, then 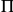 has no ptas unless 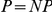.

We refer to Ausiello *et al.* [11] for further reading.

#### Parameterized Complexity

A parameterized problem is a (decision) problem equipped with an additional input called the *parameter*. Typically the parameter will numeric and should be independent of the size of the instance and relatively small. A problem 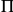 is *fixed-parameter tractable* if there is an algorithm that solves the problem in time bounded by 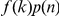 where 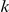 is the parameter, 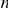 is the size of the input, 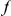 is a computable function and 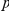 is a polynomial.

As we do not require the parameterized notion of hardness, we refer the reader to Flum and Grohe [12] for complete coverage.

## Results and Discussion

### The Complexity of AH-Cut

We first turn to theoretical results for AH-Cut. We show that the optimisation version of the problem is 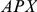-hard, and consquently that the decision version is 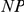-complete, indicating that AH-Cut is not has no polynomial time algorithm, but has no polynomial time approximation scheme, under standard complexity theoretic assumptions. Under the parameterized complexity framework however we show that AH-Cut is fixed parameter tractable with a 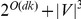 time algorithm.

### NP-Completeness and APX-Hardness

We demonstrate the 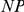-completeness for AH-Cut via an 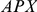-hardness reduction from Max-Cut which is known to be 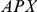-hard [3] and 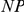-complete [13].

Max-Cut

*Instance:* A graph 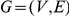, a positive integer 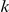.

*Question:* Is there a set 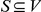, where 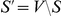 such that 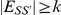
?

The goal of the optimisation version of Max-Cut is to maximise 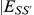.

Let 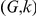 be an instance of Max-Cut with 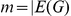 and 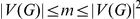 (we may assume that there is at least one cycle, as the maximum cut of any forest is trivially 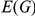), we construct an instance 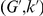 of AH-Cut where 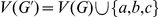 and 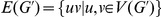 (i.e., 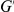 is a complete graph). The elements of 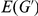 are weighted as follows: if 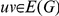, then we set 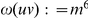, if 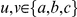 we set 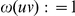 and for all other edges 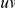 we set 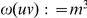. We set 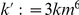. Clearly we can obtain 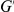 in polynomial time.

Before moving to the hardness proof, we first prove some auxilliary lemmas.

#### Lemma 1

*Let* 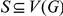 *and* 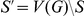 *where* 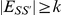 *, then* 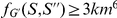 *where* 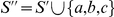 *.*

*Proof.* Consider the objective function
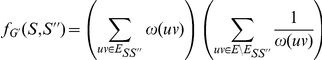
and let 
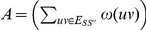
 and 
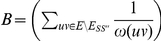
.

Each of the edges between 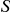 and 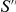 that are also in 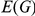 contribute 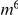 to 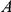, and all other edges that are also in 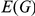 contribute 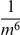 to 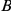. As 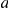, 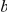 and 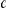 are in 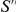, the edges 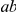, 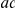 and 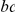 contribute 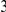 to 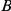. The edges between 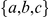 and 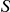 contribute 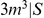 to 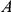. Therefore
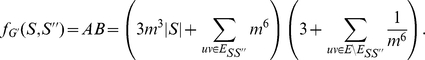
As 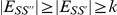, 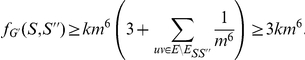



#### Lemma 2

*Assume* 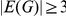 *. Let* 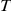 *be a subset of* 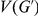 *and* 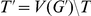 *. If* 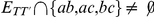 *then* 
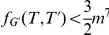
.

*Proof.* Assume 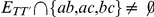, without loss of generality (by switching 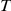 and 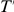) we may assume that 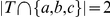. Let 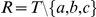, 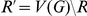 and 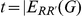. Let 
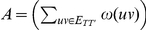
 and 
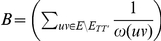
.

As 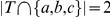 we know 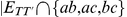, which contributes 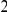 to 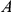. The edges in 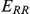 contribute 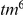 to 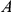. As two vertices from 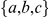 are in 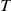, the edges between those two vertices and 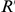 contribute 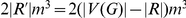 to 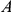. The third vertex in 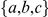 contributes 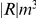 to 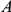.

One of the edges of 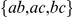 is not in 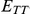 and thus contributes 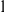 to 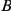. There are 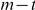 edges in 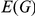 that are not in 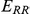 (and thus not in 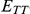) and so contribute 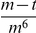 to 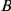. The edges between the two 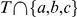 vertices and 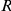 contribute 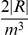 to 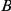. Finally the edges between the 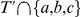 vertex and 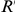 contribute 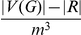 to 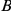. Thus in total we have
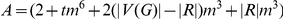
and
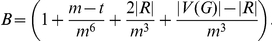
As 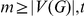 and 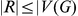 we have
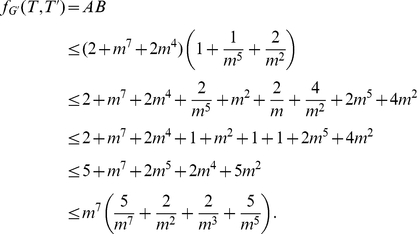
As we assume that 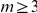, 
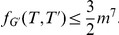



#### Lemma 3

*Assume* 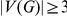 *. Let* 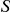 *be a subset of* 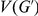 *and* 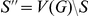 *such that* 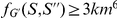 *. In polynomial time we can obtain an* 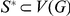 *such that* 
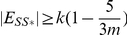
 *.*

*Proof.* If 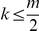 we may apply the greedy algorithm of Mahajan and Raman [14] which returns a set 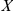 such that 
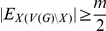
. Therefore we may take 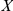 as 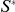 and we have 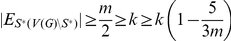.

If 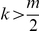, we have that 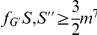. Then by Lemma 2, 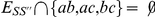. Without loss of generality we may assume that 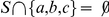 (by switching 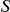 and 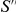 as necessary). Denote 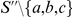 by 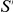. As 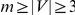 we have 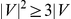. We also have that 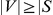. We may then observe that
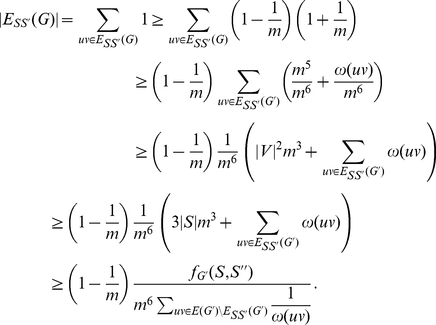
We know that 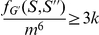, and that 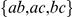 contributes 3 edges to 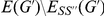, as 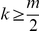 there are at most 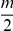 edges of 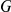 that are in 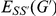 and there are at most 
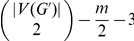
 edges otherwise unnaccounted for in 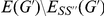. Therefore:
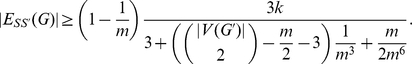
As 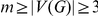:
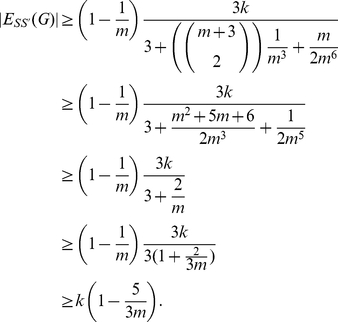



We are now prepared to prove the main theorem of this section.

#### Theorem 4

AH-Cut *is* 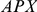 *-hard and* 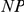 *-complete.*

*Proof.* Assume there is a 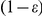-approximation algorithm 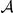 for AH-Cut. We show that this implies a 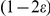-approximation algorithm for Max-Cut. Let 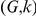 be an instance of Max-Cut and 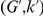 be the corresponding instance of AH-Cut derived from the reduction described above. If 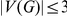 or 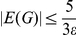, we solve the instance by complete enumeration in constant time. Otherwise assume the optimal cut of 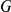 cuts at least 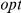 edges of 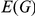 and induces the partition 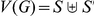. By Lemma 1 the partition 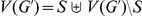 induces a solution for AH-Cut such that 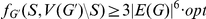. Algorithm 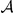 will give a solution with 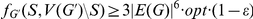. Then by Lemma 3 we have a set 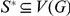 such that
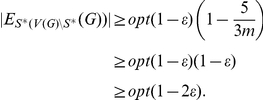



It is clear that AH-Cut is in 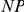. Given a partition, we simply calculate 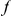 for that partition and compare to the target value.

### Fixed-Parameter Tractability

We show that AH-Cut is fixed-parameter tractable via a greedy localisation technique. First we compute a greedy solution as follows:

1. Pick an edge 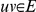 such that 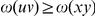 for every 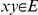. Add 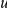 to 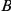 and 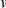 to 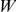.
2. While 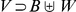 do
  - Pick a vertex 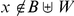 such that 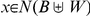.
  - If 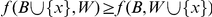 then set 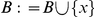, otherwise set 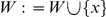.

Note that we assume that 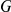 is connected. If 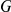 is not connected then all vertices of degree 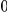 can be discarded, and the initial selection of vertices must take an adjacent pair from each connected component, then the algorithm continues as before. After all vertices have been assigned if 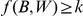, then we answer Yes. If 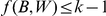 we make the following claim:

#### Lemma 5

*Let* 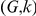 *be an instance of* AH-Cut *and* 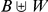 *a partition of* 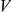 *such that* 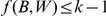 *, then* 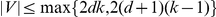 *and* 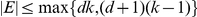 *.*

*Proof.* Let 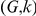 be such an instance and 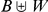 the partition.

If 
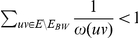
, then in particular we know that 
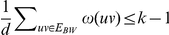
, therefore 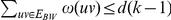 and we have 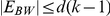. Furthermore if 
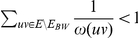
, then there are at most 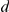 edges in 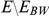. Thus the total number of edges is at most 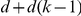 and we have at most 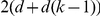 vertices in the graph.

If 
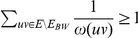
, then we immediately have that 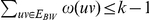 and therefore 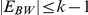. The case with the most edges with both endpoints in the same partite set is then when there is only one edge between the two partite sets, therefore 
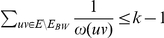
, then 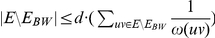, therefore 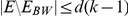 and there are at most 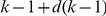 edges and 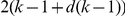 vertices in the graph.

As the instance is bounded by a function of 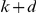, we can exhaustively search the instance in time 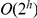 where 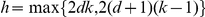.

This algorithm immediately gives the following result:

#### Theorem 6

AH-Cut *is fixed-parameter tractable with an algorithm running in time* 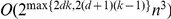 *where* 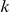 *is the optimisation target value,* 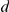 *is the maximum edge weight and* 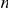 *is the number of vertices in the input graph.*

### AH-Cut in Practice

We apply our algorithm to four datasets: (i) melanoma-colon-leukemia data from National Cancer Institute, U.S [15] (involving gene expression of 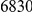 genes for 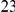 samples); (ii) SARS data of Yap *et al.* [16] and (iii) tissue type data given by Su *et al.* [17] (involving gene expression of 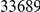 genes for 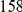 tissue samples).

We also consider a large synthetic dataset consisting of 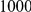 samples and 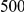 features with a known optimal solution with three clusters. Despite the size of this datasets, our algorithm finds all three clusters.

In each case we compare our algorithm to Normalized-Cut and where possible to 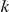-Means and Graclus. Implementation details for the memetic algorithm are given in the Materials and Methods section.

### Melanoma-Colon-Leukemia data from NCI

**Figure 1:**
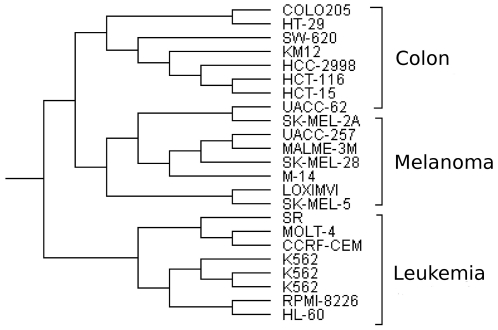
Dendrogram generated from AH-Cut for the melanoma-colon-leukemia dataset.

For the first comparison we use a subset of the data for 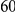 cancer samples taken for the National Cancer Institute's (NCI) screening for anti-cancer drugs [15]. The dataset consists of 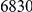 gene expressions of 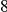 melanoma, 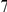 colon tumour and 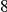 leukaemia samples. The reason for taking these three sets of samples is that others (non-small cell lung cancer, breast cancer, etc.) have heterogeneous profiles and removing these gives an expected solution of three clear clusters. Laan and Pollard [18] show that this simple dataset is already hard to cluster using agglomerative hierarchical clustering methods. Nevertheless, AH-Cut is able to cluster the samples of these three diseases effectively, see Figure 1 for the whole dendrogram generated by AH-Cut. We use centred correlation distance as the distance metric to maintain consistency with Golub *et al.* [19].

Conversely, Normalized-Cut behaves inconsistently in allocating samples to the partitions. Using the negated distance matrix as a similarity matrix and choosing two clusters, it either separates melanoma from colon and leukemia samples, or leukemia from colon and melanoma samples, or splits the leukemia sample group. Even when number of clusters is specified as 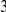, leukemia samples are split between different clusters. Using 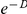 as the similarity matrix, where 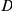 is the distance matrix, gives worse results.

On the other hand, 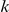-Means performs much better than Normalized-Cut and successfully separates melanoma from colon and leukemia samples when 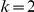 and gives three distinct clusters of colon, melanoma and leukemia samples when 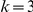.

### SARS

**Figure 2:**
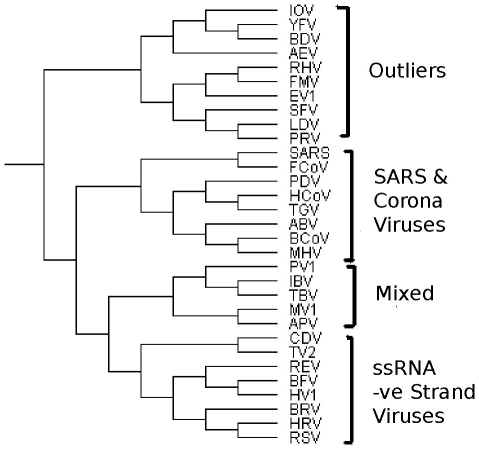
Dendrogram generated by AH-Cut for the SARS dataset.

Next we analyse Yap *et al.*'s [16] dataset for Severe Acute Respiratory Syndrome (SARS). To explore the exact origin of SARS, the genomic sequence relationships of 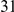 different single-stranded RNA (ssRNA) viruses (both positive and negative strand ssRNA viruses) of various families were studied. Yap *et al.* [16] generate the tetra-nucleotide usage pattern profile for each virus from which a distance matrix based on correlation coefficients is created. We use this distance matrix for the following performance comparison of AH-Cut and Normalized-Cut. See Figure 2 for the dendrogram generated by AH-Cut for this dataset. It is interesting to note that SARS virus is grouped in the same subtree as other corona viruses and is closest to the Feline Corona Virus (FCoV). Notice that these are all positive strand ssRNA viruses. This group of SARS and Corona viruses also contains other viruses (Porcine epidemic diarrhoea virus (PDV), Transmissible gastroenteritis virus (TGV), Avian infectious bronchitis virus (ABV), Murine hepatitis virus (MHV)). There is also a group of positive strand ssRNA viruses, called “Outliers”, which exhibit differences in their tetra-nucleotide usage pattern from the rest. Yellow Fever Virus (YFV), Avian Encephalomyelitis Virus (AEV), Rabbit Hemorrhagic disease Virus (RHV), Equine arteritis Virus (EV1), Lactate Dehydrogenase-elevating Virus (LDV) were also identified as outliers by Yap *et al.* [16]. This group also includes other ssRNA positive strand viruses - Igbo ora virus (IOV), Bovine viral diarrheoa (BDV), Foot and mouth disease virus C (FMV) and Simina Haemorrhagic fever virus (SFV). The negative strand ssRNA viruses are clustered in two subgroups, one unmixed with the positive strand ssRNA viruses, the remainder in the group “Mixed”. The first class (called -ve strand ssRNA viruses in Figure 2) covers Canine Distemper Virus (CDV) and Tioman virus (TV2), Reston Ebola Virus (REV), Bovine Ephemeral Fever Virus (BFV), Hantaan Virus (HV1), Bovine Respiratory syncytial Virus (BRV), Human Respiratory syncytial Virus (HRV) and Respiratory Syncytial Virus (RSV).

Normalized-Cut mixes positive and negative strand ssRNA viruses even in the first partition for the majority of runs. Graclus puts corona viruses in different partitions even when the number of partition specified is 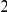. As Graclus requires a distance matrix of integers we scaled the dataset's distance matrix by 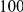 to obtain an integral distance matrix. As only a distance matrix was available, 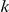-Means was not applicable.

### Tissue dataset

Next we present results of applying AH-Cut to Su *at al.*'s [17] human tissue dataset. This dataset consists of 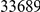 tissue specific genes from 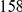 samples collected from 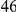 individuals. The known origin of tissue samples gives a great advantage in validating clusterings of this dataset. For brevity we will compare only the first partitioning of this dataset generated by the different algorithms. The first partition of AH-Cut consists of:

- brain related tissues;
- eye related tissues;
- face related tissues;
- testis tissues;
- others (including ovary tissue).

The second partition of AH-Cut consists of:

- bonemarrow related cells;
- blood cells;
- heart related cells;
- fœtal cells;
- others (including ovary tissue, uterine tissue and uterine corpus tissue).

The partitioning for this dataset is quite reasonable except occurrence ovary tissues in different partitions. This can be due to the possible outlier nature of ovary tissues. However, Normalized-Cut repeatedly separates the brain related tissues and thus performs even worse. Graclus performs similarly to Normalized-Cut on this dataset.


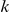
-Means agrees very well with AH-Cut in the partitions, except that it clusters placental tissues within the second partition instead of the first and it puts the two uterine tissues in different partitions. 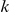-Means also puts the two ovary tissues in two different partitions.

### Synthetic large-sampled gene expression data

To test the scalability of our algorithm, we show the results of AH-Cut applied to a large synthetic dataset. Consider 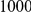 samples of 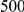 synthetic gene expression profiles corresponding to three subtypes of some disease, giving a known optimum clustering with three clusters. To generate the data, we follow Laan and Pollard's [18] method. We sample three groups of 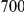, 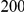 and 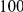 samples respectively from three multivariate normal distributions with diagonal covariance matrices, which differed only in their mean vector. The number of samples are chosen keeping in mind that in general there are some predominant subtypes of a disease and some rarer subtypes. All genes had common standard deviation 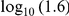, which corresponds to a 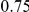-quantile of all standard deviations in an actual data set. For the first subpopulation, the first 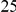 genes had a mean of 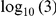, genes 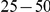 had mean of 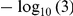, and the other 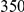 genes had mean zero. Then for the second subpopulation, genes 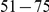 had mean of 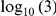, genes 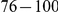 had mean of 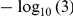 and the other 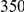 genes had mean zero. For the third subpopulation, genes 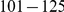 had mean of 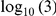, genes 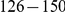 had mean of 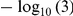 and the other 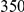 genes had mean zero. In other words, signature of the three types of cancer is related to 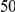 genes of which 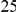 are under-expressed and 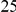 are over-expressed.

The application of AH-Cut on this dataset first separates the first group from the rest. A second application on the rest of the samples yields the second and third group as the two partitions.

When number of clusters is specified as two, Normalized-Cut clusters the first and third subtypes together and the second subtype separately. However, specifying number of clusters as three creates a partitioning which does not correspond to the expected known grouping.


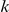
-Means puts the first subtype in one partition and the other two subtypes in another partition when 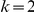, and separates three subtypes successfully when 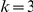.

### Conclusion

We have introduced a novel objective function for clustering based on graph partitioning. We show that the resulting problem AH-Cut is, unfortunately, 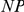-complete and 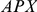-hard, but is however fixed-parameter tractable.

We then gave several test cases demonstrating the potential of the approach using a memetic algorithm. The performance of AH-Cut based clustering exceeds the performance of Normalized-Cut based clustering across a wide variety of datasets, including large scale datasets, and notably datasets with known optimal clusterings. AH-Cut based clustering also has a wider applicability than 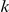-Means based clustering, and at least equal performance.

There are several avenues for further research from this initial exploration. The fixed-parameter tractability of AH-Cut promises the possibility of a practical *exact* algorithm, which would give stronger evidence of AH-Cut's performance, as random elements would be removed.

Further studies on datasets of all kinds would also be useful to explore the strengths and weaknesses of AH-Cut based clustering, especially in comparison to other existing methods.

Tangentially, the quality of the memetic algorithm solutions suggest that there may be a link between the fixed-parameter tractability and the performance of the memetic algorithm. As established by the fixed-parameter tractability of AH-Cut, if a simple greedy algorithm does not produce a solution with a sufficiently high objective value, then the instance size must be bounded by an relatively simple function of the parameters. Therefore it is possible that under these conditions the local search component of the memetic algorithm approximates an exhaustive search, or at least has a greater effectiveness. A definite link of this kind would be an interesting development for both parameterized complexity and memetic algorithmics, above and beyond this application.

## Materials and Methods

The complexity analysis of the AH-Cut problem employ standard complexity theory techniques [11], [12].

The NCI60 cancer dataset was drawn from the NCI60 anti-cancer drug screening program data [20] and the gene expression data for the cell lines was given by Ross *et al.* [15].

The tissue dataset is drawn from Su *et al.* [17].

The synthetic dataset was generated using the methods of Laan and Pollard [18].

For the comparisons we use Gene Cluster's implementation of 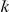-Means [4] (http://bonsai.ims.u-tokyo.ac.jp/~mdehoon/software/cluster/software.htm#ctv), Dhillon *et al.*'s Graclus software [9] (http://userweb.cs.utexas.edu/users/dml/Software/graclus.html) and Shi and Malik's implementation of Normalized Cut [1].

All experiments were performed on a Dell laptop with a 1.67GHz processor and 2GB of RAM with the software written in Java.

### A memetic algorithm for AH-Cut

We have implemented AH-Cut via a memetic algorithm [21], [22]. Memetic algorithms provide a population-based approach for heuristic search in optimization problems. Broadly speaking they combine local search heuristics with crossover operators used in genetic algorithms [23]–[25]. The essence of our algorithm is similar to the work of Merz and Freisleben [26] for Graph Bi-partitioning. Differences arise from the fact that we need to remove the constraint of equal partitioning of the graph. The method consists of three main procedures: a greedy algorithm for initialization of a set of solutions for AH-Cut (detailed in the parameterized algorithm); a differential greedy crossover for evolution of the population; and a variable neighborhood local search, influenced by Festa *et al.* [27], to improve the newly generated solutions.

We use a ternary tree for population similar to Buriol *et al.* [28] and keep two solutions at each node of this tree. One solution is the best obtained so far at the node, called *pocket solution* and the other one is the *current* solution. Essentially, if we generate a current solution by recombination or local search which is better than the pocket solution, we swap the current solution with the pocket solution. Furthermore, each parent node of the tree must have better pocket solution than its children's pocket solutions. Similar tree structures were previously employed successfully for various combinatorially hard problems [28]–[30].

#### Differential Greedy Crossover

We allow a crossover of a parent's pocket solution with a child's current solution to ensure the diversity in the population. All vertices that are contained in the same set for both the parents, are included in the same set in the offspring. Then both sets are filled according to a greedy recombination method similar to the greedy algorithm used for the parameterized algorithm. Suppose, the parent solutions 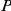 and 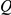 have the partitions 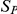, 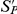 and 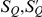 respectively (after interchanging the sets suitably). Then the starting set 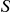 (resp. 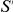) for the offspring is given by the intersection 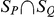 (resp. 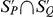), with the remainder of the partition calculated greedily.

#### Local Search

We employ a *variable-neighborhood search (VNS)*, first proposed by Hansen and Mladenovic [31] for a local search in the neighborhood of the new offspring. Contrary to other local search methods, VNS allows enlargement of the neighborhood structure. A 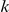-th order neighbor of a paritition giving a solution 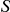 for AH-Cut is obtained by swapping the partite set of 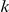 vertices. In VNS, first local search is done starting from each neighbor 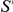 of the current solution 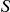. If a solution 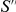 is found which is better than 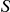, then the search moves to the neighborhood of 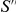. Otherwise, the order 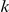 of the neighborhood is increased by one, until some stop criterion holds. We use maximum value of 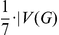 for 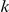.

#### Diversity

Whenever the population stagnates (fails to improve the objective value), we keep the best solution and re-initialize the rest of solutions (using the greedy algorithm with a randomised starting vertex pair) in the set and run the above process again for certain number of generations (say, 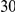).

To get the optimal solution for very small sized problems (graphs containing less than 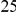 vertices), we used backtracking. Notice that even though backtracking gives us an optimal solution, a greedy or memetic algorithm may not. By applying this method (backtracking, memetic or greedy algorithm depending on the number of vertices) recursively, we have at each step a graph as input, and the two subgraphs induced by each of the sets of the vertex partition as output; stopping when we arrive to a graph with just one vertex, we generate a hierarchical clustering in a top-down fashion.
